# Inflammatory myofibroblastic tumor in the liver: a case report

**DOI:** 10.3389/fonc.2024.1349692

**Published:** 2024-05-28

**Authors:** Yinying Meng, Jinlan Xie, Yan Liang, Mulan Wu, Yi Lu, Qian Lu

**Affiliations:** ^1^ Department of Radiology, Wuzhou Gongren Hospital, Wuzhou, China; ^2^ Department of Ultrasound, The University of Hong Kong Shenzhen Hospital, Shenzhen, China; ^3^ Department of Pathology, Wuzhou Gongren Hospital, Wuzhou, China

**Keywords:** hepatic inflammatory myofibroblastic tumor, hepatectomy, histology, imaging, case report

## Abstract

**Background:**

Hepatic inflammatory myofibroblastic tumor (IMT) is an infrequent tumor with potential malignancy. However, it lacks specific clinical symptoms and usual imaging features.

**Case presentation:**

A 34-year-old woman had a six-month history of fever and on-and-off pain in the upper right part of her abdomen that lasted for two weeks. Imaging tests revealed a liver mass initially thought to be liver malignancy, but subsequent histopathological examination after liver removal confirmed the diagnosis as hepatocellular inflammatory myofibroblastic tumor (HIMT).

**Conclusion:**

Hepatic inflammatory myofibroblastic tumor (IMT) is an uncommon growth with vague clinical symptoms and lab results. Surgical removal remains the primary treatment method, resulting in favorable prognostic outcomes.

## Introduction

Inflammatory myofibroblastic tumor (IMT) of the liver, previously known as inflammatory pseudotumor (IPT), has been identified in various somatic and visceral locations, although it was originally described in the lungs. IMT has been recognized as a distinct pathological entity separate from the broader category of IPTs (1, 2). This study aims to conduct a comprehensive literature review to investigate and consolidate the unique and shared characteristics of this uncommon HIMT case. The objective is to provide healthcare professionals with the tools to accurately diagnose the condition based on its defining traits, explore different treatment approaches, and assist patients in achieving optimal outcomes and a favorable prognosis.

## Case description

A 34-year-old woman sought medical care on September 21, 2022, due to persistent fevers over a six-month period. The fevers were of unknown origin but responded transiently to oral antipyretics. In the past two weeks, she started to experience a dull pain in the right upper quadrant of her abdomen, worsening when turning from lying on her back to her left side. These pain episodes were brief, lasting only a few seconds. Additionally, she reported a significant loss of appetite. Her medical history was unremarkable, with no major conditions such as hepatitis B or C. Physical examination revealed tenderness in the liver area upon percussion and visible superficial veins across the abdomen, without other notable abnormalities. Some laboratory indicators showed abnormalities, as listed in **Table 1**. Notably, alpha-fetoprotein (AFP), carcinoembryonic antigen (CEA), cancer antigen 15–3 (CA15–3), and CA-199 all yielded normal results.

**Table 1:** Abnormal laboratory indicators.

Test item	Result	Unit	Reference range
Hemoglobin	76	g/L	110–150
white blood cell count	11.52	10^9/L	3.50–9.50
The percentage of lymphocytes	11.1	%	20.0–50.0
The percentage of eosinophils	18.4	%	0.4–8.0
D-Dimer	5.374	μg/L	0.000–0.550
CA-125	87.8	U/ml	0.00–35.00

**Figure 1:**
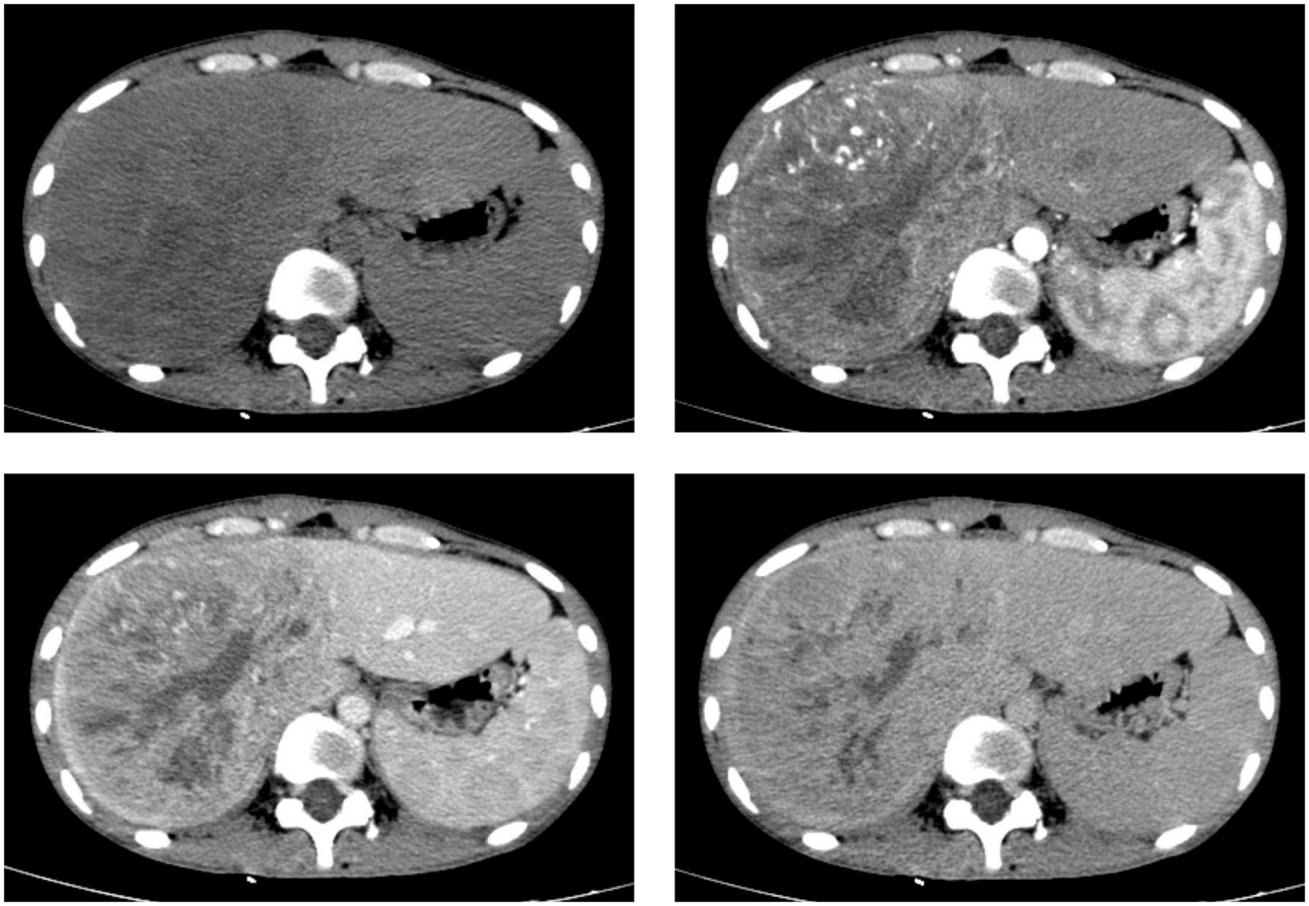
CT scan images in axial views before and after the injection of a contrast agent depict a sizable liver mass with well-defined boundaries. The mass exhibits heterogeneous enhancement in both the hepatic arterial and portal venous phases, although the enhancement is less intense than the normal liver parenchyma.

**Figure 2:**
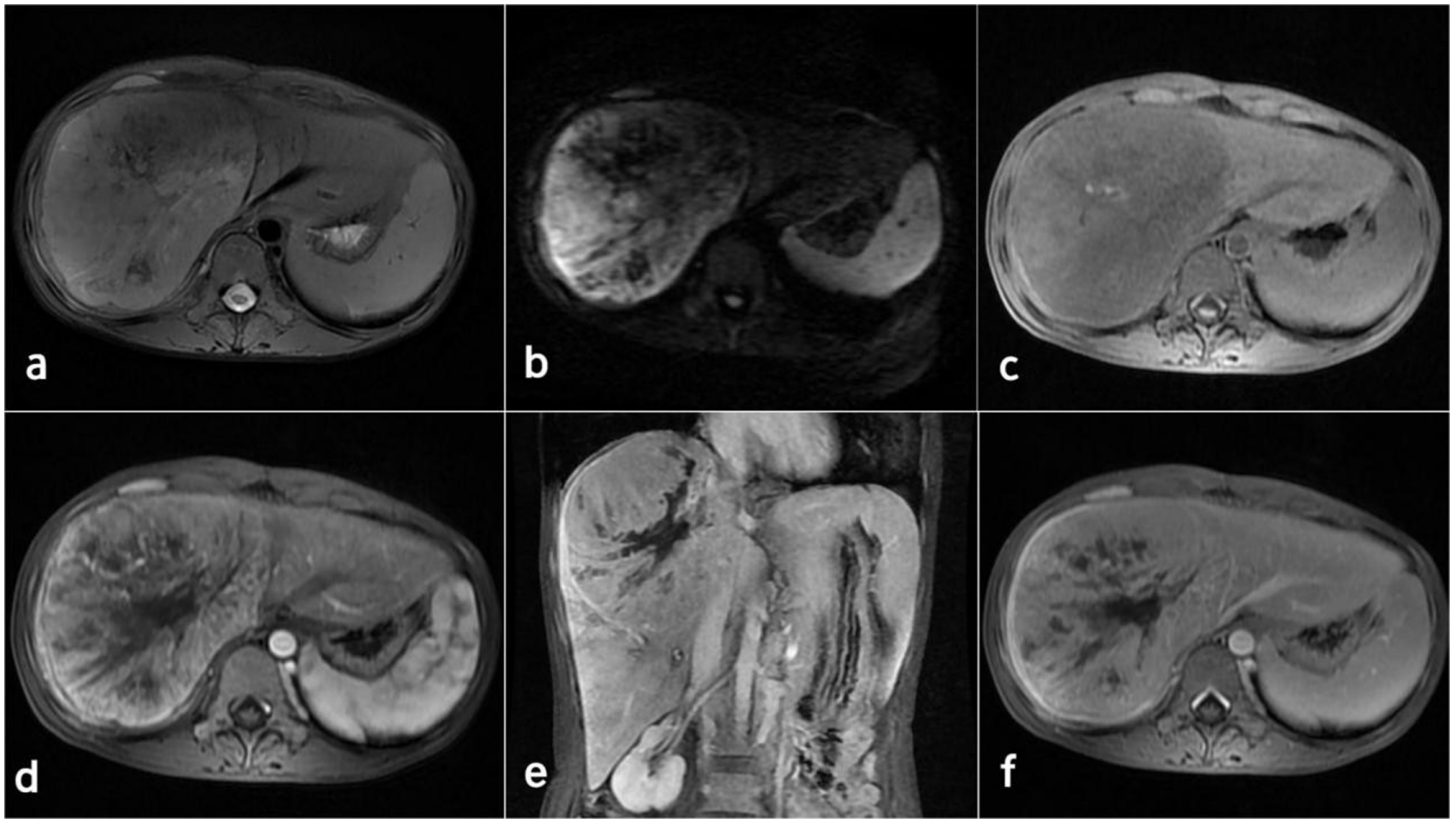
MRI images of the inflammatory myofibroblastoma of the liver. **(A)** T2WI. **(B)** DWI (b=800). **(C-F)** display the enhanced scan images.

Contrast-enhanced computed tomography (CT) and magnetic resonance imaging (MRI) revealed the presence of a sizable liver mass, raising suspicion of giant cell carcinoma (GCC) (**Figures 1**, **2**). The CT scan showed a significant, irregular hypodense mass measuring approximately 14.2 cm x 10.4 cm x 12.9 cm in the right hepatic lobe. The mass exhibited a parenchymal CT value of around 30 Hounsfield units (Hu). During the arterial phase of the enhanced scan, it displayed noticeable irregular enhancement with a CT value of approximately 45 Hu. The parenchymal part of the lesion demonstrated progressive enhancement during the portal venous and equilibrium phases, with CT values ranging from about 50 to 65 Hu. Moreover, areas of irregularity were visible within the lesion, which did not exhibit any enhancement. On T1-weighted imaging (T1WI), a slight decrease in signal intensity was observed along with punctate low signal shadows. T2-weighted imaging with fat suppression (T2WI+FS) revealed mixed signal shadows with both high and low signal intensities. On diffusion-weighted imaging (DWI) with a low b-value, a slight increase in signal intensity was noticed. However, as the b-value increased, there was no significant signal attenuation within the lesion, maintaining high signal intensity. Small patchy low signal shadows were also visible within the lesion at high b-values. During the arterial phase, the enhancement scan showed uneven enhancement, with a decrease in enhancement during the portal and delayed phases. Multiple irregular non-enhancing areas were also identified within the lesion.

A partial hepatectomy was performed following consultation with the patient and her family. The excision of the mass was successfully carried out. Intraoperative observations confirmed the absence of malignancy in the peritoneum and other organs. However, a substantial tumor measuring approximately 15 cm x 12 cm x 12 cm was predominantly found in the right lobe of the liver, leading to compression of the inferior vena cava, hepatic veins, and hepatic artery. The remaining liver surface appeared normal without any nodules.

## Diagnostic assessment

Immunohistochemistry results indicated the following: AE1/AE3 (-), Glypican3 (-), Hepatocyte (-), Ki-67 (+, 50%), Vimentin (+), STAT6 (-), SMA (partially +), MPO (-), Desmin (partially +), Myogenin (-), MyoD1 (-), Actin (-), CD45 (+, background inflammatory cells), CD3 (T cells), CD5 (T cells), CD79a (B cells), CD20 (B cells), CD23 (B cells), ALK (-), CD15 (sporadic +), CD30 (-), IgG4 (-), CD4 (T cells), CD8 (T cells), CD68 (+), S-100 (partially +), IgG4 (-), CD34 (small amount +), Dog-1 (-). These immunohistochemical findings were consistent with an IMT.

Staining results were as follows: Periodic Acid-Schiff staining was negative, fungal fluorescent staining showed no presence of fungi, acid-fast staining did not reveal any acid-fast organisms, and acid-fast fluorescent staining did not detect any acid-fast organisms.

**Figure 3:**
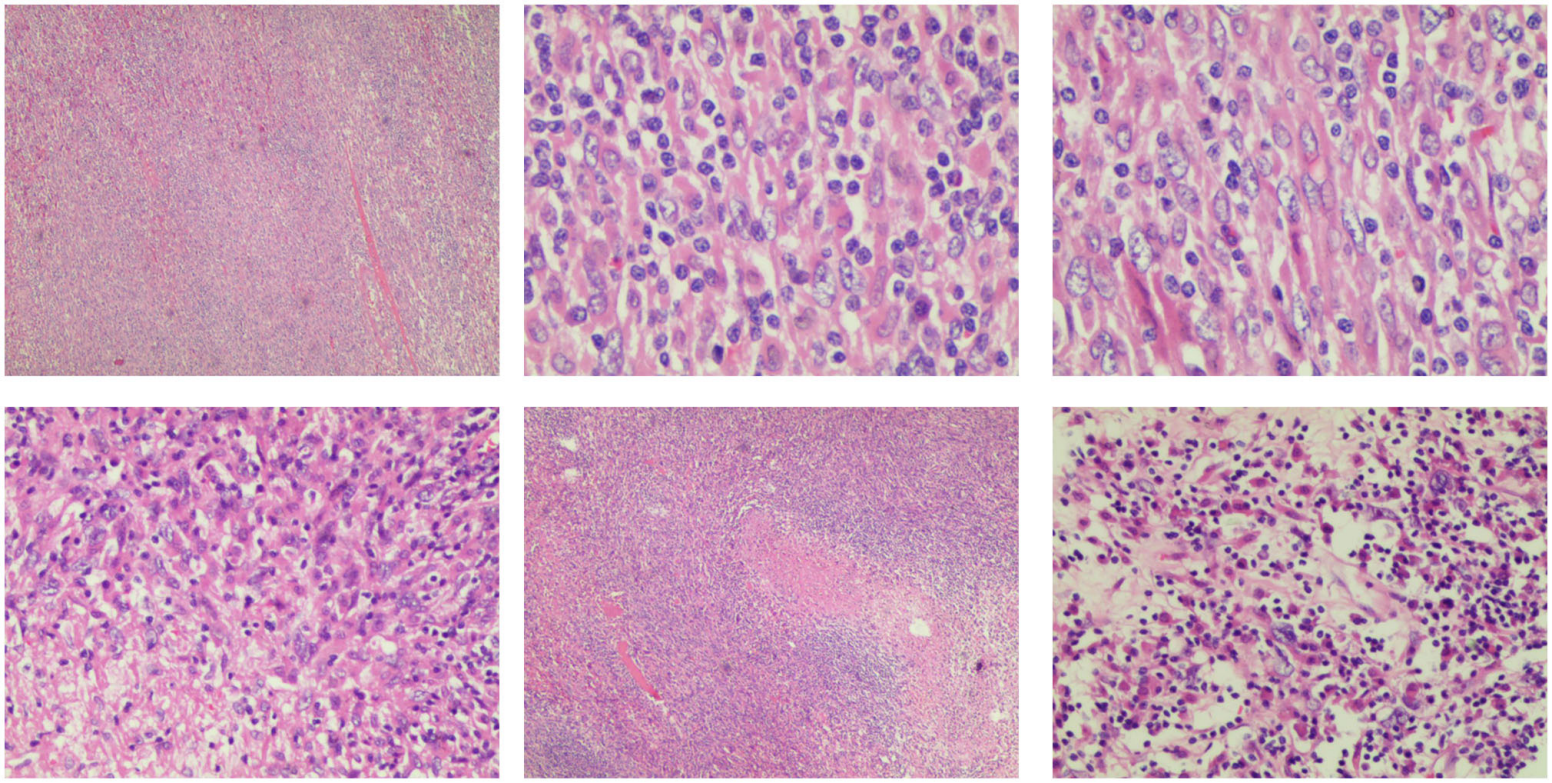
Pathological image of inflammatory myofibroblasts in the liver. The tumor is composed of a proliferation of spindle cells and a large number of inflammatory cells, predominantly T lymphocytes. Collagen fiber proliferation is evident, accompanied by focal necrosis. No significant mitotic figures are observed.

Microscopic examination of the excised tumor revealed the presence of spindle cells and a significant population of T lymphocytes, accompanied by collagen fiber proliferation and localized necrosis. No significant mitotic figures are observed (**Figure 3**). Combined with morphology and immunohistochemistry, the pathologist diagnosed it as HIMT.

## Follow-up and outcomes

The patient achieved a successful discharge with good general health and normalized laboratory values. We recommended continued monitoring through regular outpatient clinic visits. The most recent follow-up in March 2024 revealed no evidence of recurrence based on normal tumor markers and negative findings on contrast-enhanced computed tomography. Notably, the patient demonstrated excellent adherence to the follow-up schedule, attending all appointments without any complications.

## Discussion

Inflammatory Myofibroblastic Tumor (IMT) (3) is a unique and rare mesenchymal tumor that has been previously referred to as inflammation-like disease, plasma cell sarcoma, and other names. Microscopic examination of IMT may reveal various histological patterns, including a myxoid vascular pattern characterized by loosely arranged spindle or stellate-shaped cells in a myxoid edematous matrix, a compact pattern with cellular fascicles or storiform bundles, and a hypocellular fibrous pattern with dense collagen (4). IMT typically predominantly presents one subtype, although other subtypes may also be present. It can manifest in various parts of the body, but it is uncommon in the liver (5). Liver IMT is a rare, relatively benign, and atypical condition of the liver characterized by local chronic non-specific inflammatory cell infiltrates and fibrous connective tissue nodules. The etiology of liver IMT may involve microbial infection of hepatocytes, leading to acute effusion and progressive atypical hyperplasia, or inflammation resulting from portal vein infection leading to pylephritis, or macrophages surrounding the focus of liver abscesses causing fibrosis or vitrification, resembling inflammation-like disease. Chronic cholecystitis, fibrosis of the bile duct wall, and other related conditions can arise due to recurrent bile stasis (3, 5–7). Liver IMT does not exhibit age-related variations (8), and its clinical manifestations are generally subtle and lack significant specificity (8, 9). Some patients may experience symptoms such as abdominal pain and fever, while tumor markers typically yield negative results. In this case report, Ca-125 levels were slightly elevated, but further investigation is needed to establish its correlation with IMT.

IMT primarily occurs in the peripheral or subcapsular regions of the liver, with the majority of cases featuring low-density lesions on CT scans, and in rare instances, isodense lesions may be observed. On T2-weighted images, the predominant patterns include homogeneous or inhomogeneous signal hyperintensity and a targetoid appearance characterized by a hyperintense core. On MR T1-weighted images, lesions usually appear hypo- or isointense, in line with previous research findings (10–12). The portal vein serves as the primary source of blood supply to IMT, with minimal contribution from the hepatic artery. During the hepatobiliary phase, the lesion demonstrates hypointensity, indicating the absence of functional hepatocytes (12). In this specific case report, it was noted that the portal phase exhibited a higher degree of enhancement compared to the arterial phase, suggesting the presence of delayed enhancement, consistent with previous reports.

Researchers previously considered IMTs to represent the neoplastic subset of the family of inflammatory pseudotumors. This umbrella term encompasses spindle cell proliferations of uncertain histogenesis with a variable inflammatory component. Therefore, many researchers used the terms “inflammatory myofibroblastic tumor” and “inflammatory pseudotumor” interchangeably (13). Distinguishing between inflammatory myofibroblastic tumors (IMTs) and inflammatory pseudotumors requires a multi-modal approach due to overlapping clinical presentations. Histopathology reveals IMTs with fibroblast and myofibroblast hyperplasia alongside chronic inflammation, while inflammatory pseudotumors may show epithelioid cells, mucinous matrix, and intercellular inflammatory infiltrates. Desmin immunohistochemistry can be positive in IMTs, aiding differentiation. Emerging evidence suggests distinct genetic alterations between the two, offering a promising avenue for future diagnostic refinement (14).

The differential diagnosis of liver IMT should also include the following six diseases: Hepatocellular carcinoma (HCC), which may share similar enhancement patterns with IMT, can be differentiated by atypical HCC, alpha-fetoprotein (AFP) positive, history of hepatitis B and cirrhosis, and the presence of a pseudocapsule on imaging. Cholangiocarcinoma also has delayed enhancement but usually shows dilated bile ducts around the tumor and serum CA19–9 elevation (15). Liver abscess may have wall enhancement resembling IMT, but usually accompanied by fever, pyrexia, and elevated neutrophil levels. In some cases, liver abscess may progress to IMT due to fibrosis or remodeling caused by surrounding macrophages. Hepatic peliosis may present as target-like lesion. T2-weighted images can differentiate it from IMT because peliosis often shows very high signal intensity (SI), like hemangioma. After contrast agent, peliosis may show centripetal or centrifugal enhancement (16). Hepatic epithelioid angioendothelioma is characterized by “lollipop sign” on imaging, portal vein or hepatic vein dissemination, and termination at the edge of the lesion (17).Inflammatory pseudotumor-like follicular dendritic cell tumor (IPT-like FDCT) exhibits clinical and radiographic characteristics similar to IMT, necessitating a pathological examination for definitive diagnosis. The gold standard for diagnosing IPT-like FDCT is the immunohistochemical staining that reveals CD21 and CD35 positivity, coupled with *in situ* hybridization demonstrating EBER positivity (18). Primary liver cancer was diagnosed in this case despite negative AFP results and enhancement pattern different from hepatocellular carcinoma. This may be because of limited knowledge of IMT among the diagnostic physicians and large size of the lesion that made it difficult to exclude liver cancer definitively.

Surgical resection remains the most effective treatment for IMT. The choice of the appropriate surgical approach should be based on factors such as the tumor’s location, size, and extent of involvement, with the goal of achieving complete removal. If nearby organs are involved, they should also be removed. When the patient’s overall health is good, surgical resection can be expanded as needed. After surgery, close follow-up observation is essential. The lack of multicenter and large sample clinical research has led to a lack of consensus on treatments for non-resectable and incompletely resected IMT. Alternative treatment options include non-steroidal anti-inflammatory drugs, especially COX-2 inhibitors, steroid hormones, radiation therapy, chemotherapy, and crizotinib (19). IMT tends to have a certain tendency to recur, with higher recurrence rates observed in the head and neck, paranasal sinuses, mesentery, and retroperitoneum (20). When postoperative symptoms reoccur, it suggests tumor recurrence. For recurrent cases, surgery may be attempted again to alleviate the condition. Factors such as patient age, tumor size, tumor location, and the completeness of the initial surgical resection are related to local recurrence.

In summary, hepatic inflammatory myofibroblastic tumor is a rare neoplasm characterized by non-specific clinical symptoms and laboratory findings. Detecting lesions situated at the liver’s periphery or subcapsular region can be helpful for diagnosing IMT. Surgical resection is often necessary to confirm the diagnosis, emphasizing the significance of preoperative biopsy to guide treatment decisions. At present, surgical resection remains the primary treatment approach, and it yields favorable prognostic outcomes. However, it’s essential to recognize the potential for local recurrence and metastasis. Therefore, long-term monitoring and surveillance are imperative.

## Patient perspective

The patient described the initial shock and disbelief upon receiving the diagnosis, as she had always considered herself healthy. She emphasized the significance of clear communication regarding treatment options, potential side effects, and lifestyle modifications. She expressed gratitude for the multidisciplinary team’s efforts in improving her understanding of the disease and facilitating her active involvement in decision-making.

## Data availability statement

The original contributions presented in the study are included in the article/supplementary material. Further inquiries can be directed to the corresponding authors.

## Ethics statement

Written informed consent was obtained from the individual(s) for the publication of any potentially identifiable images or data included in this article.

## Author contributions

YM: Conceptualization, Methodology, Project administration, Resources, Writing – original draft, Writing – review & editing. JX: Data curation, Formal analysis, Writing – original draft, Writing – review & editing. YLI: Conceptualization, Data curation, Formal analysis, Writing – original draft, Writing – review & editing. MW: Data curation, Writing – original draft, Writing – review & editing. YLU: Writing – review & editing, Resources, Conceptualization, Investigation. QL: Writing – review & editing, Conceptualization.
